# A New Parametric Life Distribution with Modified Bagdonavičius–Nikulin Goodness-of-Fit Test for Censored Validation, Properties, Applications, and Different Estimation Methods

**DOI:** 10.3390/e22050592

**Published:** 2020-05-25

**Authors:** Mahmoud Mansour, Mahdi Rasekhi, Mohamed Ibrahim, Khaoula Aidi, Haitham M. Yousof, Enayat Abd Elrazik

**Affiliations:** 1 Management Information System Department, Yanbu, Taibah University, Yanbu 46421, Saudi Arabia; Mahmoud.mansour@fcom.bu.edu.eg (M.M.); ekhalilabdelgawad@taibahu.edu.sa (E.A.E.); 2 Department of Statistics, Mathematics and Insurance, Benha University, Benha 13513, Egypt; haitham.yousof@fcom.bu.edu.eg; 3 Department of Statistics, Faculty of Mathematical Sciences and Statistics, Malayer University, Malayer 25569, Iran; rasekhimahdi@gmail.com; 4 Department of Applied Statistics and Insurance, Faculty of Commerce, Damietta University, Damietta 34519, Egypt; 5 Laboratory of probability and statistics LaPS, University Badji Mokhtar, Annaba 23000, Algeria; khaoula.aidi@yahoo.fr

**Keywords:** Bagdonavicius–Nikulin, Burr X Family, non-Bayesian methods, maximum likelihood estimation, Cramer-Von-Mises, moments, order statistics, L-moments, percentile estimation, Weibull model

## Abstract

In this paper, we first study a new two parameter lifetime distribution. This distribution includes “monotone” and “non-monotone” hazard rate functions which are useful in lifetime data analysis and reliability. Some of its mathematical properties including explicit expressions for the ordinary and incomplete moments, generating function, Renyi entropy, δ-entropy, order statistics and probability weighted moments are derived. Non-Bayesian estimation methods such as the maximum likelihood, Cramer-Von-Mises, percentile estimation, and L-moments are used for estimating the model parameters. The importance and flexibility of the new distribution are illustrated by means of two applications to real data sets. Using the approach of the Bagdonavicius–Nikulin goodness-of-fit test for the right censored validation, we then propose and apply a modified chi-square goodness-of-fit test for the Burr X Weibull model. The modified goodness-of-fit statistics test is applied for the right censored real data set. Based on the censored maximum likelihood estimators on initial data, the modified goodness-of-fit test recovers the loss in information while the grouped data follows the chi-square distribution. The elements of the modified criteria tests are derived. A real data application is for validation under the uncensored scheme.

## 1. Introduction

Focusing on one of the most popular positive probability models, the Weibull (W) distribution, in this paper, we introduce a new generalization called the Burr X Weibull (BXW). The W model was proposed in 1951 and is widely used in reliability analysis and in several different fields with different applications, see, for example, ([1]). Although it includes widely usage, a negative point of the distribution is the limited shape of its hazard function, which can only monotonically increase, decrease, or remain constant. Generally, practical problems require a wider range of possibilities in the medium risk, for example, when the lifetime data present a bathtub-shaped hazard function, such as human mortality and machine life cycles. Over several years, researchers have developed various extensions and modified forms of the Weibull distribution with different numbers of parameters. A state-of-the-art survey on the class of such distributions can be found in ([1]). Some extensions of the W distribution with more than two parameters are available in the literature, such as exponentiated W (Exp-W) ([2,3]), the additive W ([4]), the Marshall–Olkin extended W ([5]), the beta inverse W ([6]), transmuted exponentiated generatized W ([7]), Marshall–Olkin additive W ([8]), the Topp Leone generated W distribution ([9]), the exponentiated generalized W Poisson ([10]), Type I general exponential W ([11]), new four-parameter W ([12]), Burr XII W ([13]), Marshall–Olkin generalized W Poisson ([14]), odd Lindley W ([15]), Lindley W ([16]), W generalized W ([17]), new extended W ([18]), Type II general exponential W ([19]), Burr X exponentiated W ([20]), odd power Lindley W ([21]), odd Nadarajah-Haghighi W ([22]), and WW Poisson ([23]). 

In this paper and after studying the mathematical properties of the BXW model, some non-Bayesian methods, such as the maximum likelihood, Cramer-Von-Mises, percentile estimation, and L-moments, is used for estimating the model parameters, are considered. For comparing non-Bayesian methods, simulation studies are provided. The importance and flexibility of the new distribution is illustrated by means of two applications of real data sets. Uncensored applications for comparing the non-Bayesian methods are presented. The censored maximum likelihood estimation is derived and, using the approach of the Bagdonavicius–Nikulin goodness-of-fit test for the right censored validation, we propose and apply a modified chi-square goodness-of-fit test for a new model. The modified goodness-of-fit statistics test was applied for the right censored real data set. Based on the censored maximum likelihood estimators on initial data, the modified goodness-of-fit test recovered the loss in information, while the grouped data followed the chi-square distribution. The elements of the modified criteria tests are derived. Finally, a real data application for validation under the uncensored scheme is presented.

## 2. The BXW Model

The cumulative distribution function (CDF) of the two parameter W distributions is given by

$$G_{\alpha ,\beta }\left(x\right)=1-exp\left(-\alpha x^{\beta }\right),$$

where α>0 scale parameter and β>0 are shape parameters. Clearly, for α=1, the two parameter W model reduces to one parameter W model. For β=1, we get the standard exponential model. The CDF and the probability density function (PDF) of one parameter W model are

(1) $$g_{\beta }\left(x\right)=\beta x^{\beta -1}exp\left(-x^{\beta }\right){|}_{\left(\beta >0,\;x>0\right)}\;\mathrm{and}\;G_{\beta }\left(x\right)=1-exp\left(-x^{\beta }\right),$$

respectively. Depending on Equation (1), we defined and study a new lifetime model called the BXW distribution. Its main characteristic is that one shape parameter is added in Equation (1) to provide more flexibility for the generated distribution. Based on the Burr X-G (BX-G) family pioneered by [24] we constructed the two-parameter BXW model and give a comprehensive description of some of its mathematical properties. The new distribution has the advantage of being capable of modeling various shapes of aging and failure criteria. Further, the BXW model is shown to fit better than at least six other competitive models, each having the same number of parameters. We aim to attract wider applications in engineering, medicine, and other areas of research. [24] defined the CDF of the BX-G family by

(2) $$F_{\theta ,\xi }\left(x\right){|}_{\left(\theta >0,\;x\in R\right)}={\left\{1-exp\left[-\Delta_{\xi }{\left(x\right)}^{2}\right]\right\}}^{\theta },$$

where $\Delta_{\xi }\left(x\right)=G_{\xi }\left(x\right)/\left[1-G_{\xi }\left(x\right)\right]$ and $\xi =\xi_{k}=\left(\xi_{1},\xi_{2},\ldots \right)$ are parameter vectors. The CDF corresponding to Equation (2) becomes

(3) $$f_{\theta ,\xi }\left(x\right)=2\theta \frac{g_{\xi }\left(x\right)G_{\xi }\left(x\right)}{{\left[1-G_{\xi }\left(x\right)\right]}^{3}}exp\left\{-\Delta_{\xi }{\left(x\right)}^{2}\right\}{\left(1-exp\left\{-\Delta_{\xi }{\left(x\right)}^{2}\right\}\right)}^{\theta -1},$$

where θ is the shape parameter. A random variable (RV) X with PDF (3) is denoted by X~BXG(θ,ξ). By substituting Equation (1) with Equation (2), the two-parameter BXW CDF of X is given by (for x > 0):

(4) $$F_{\theta ,\beta }\left(x\right){|}_{\left(\theta ,\beta >0,\;x>0\right)}={\left(1-exp\left\{-{\left[exp\left(x^{\beta }\right)-1\right]}^{2}\right\}\right)}^{\theta }.$$

The PDF corresponding to Equation (4) is given by

(5) $$f_{\theta ,\beta }\left(x\right)=2\theta \beta x^{\beta -1}\frac{\left[1-exp\left(-x^{\beta }\right)\right]exp\left\{2x^{\beta }-{\left[exp\left(x^{\beta }\right)-1\right]}^{2}\right\}}{{\left(1-exp\left\{-{\left[exp\left(x^{\beta }\right)-1\right]}^{2}\right\}\right)}^{1-\theta }}$$

where θ and β are the shape parameters representing the different shapes of the BXW distribution. We denote a RV X with PDF (5) by X~BXW(θ,β). The reliability function (RF), hazard rate function (HRF), and cumulative hazard rate function (CHRF) of X are given by

$$f_{\theta ,\beta }\left(x\right)=2\theta \beta x^{\beta -1}\frac{\left[1-exp\left(-x^{\beta }\right)\right]exp\left\{2x^{\beta }-{\left[exp\left(x^{\beta }\right)-1\right]}^{2}\right\}}{{\left(1-exp\left\{-{\left[exp\left(x^{\beta }\right)-1\right]}^{2}\right\}\right)}^{1-\theta }},$$

$$R_{\theta ,\beta }\left(x\right)=1-{\left(1-exp\left\{-{\left[exp\left(x^{\beta }\right)-1\right]}^{2}\right\}\right)}^{\theta },$$

$$h_{\theta ,\beta }\left(x\right)=\frac{2\theta \beta x^{\beta -1}\left[1-exp\left(-x^{\beta }\right)\right]exp{\left\{2x^{\beta }-{\left[exp\left(x^{\beta }\right)-1\right]}^{2}\right\}}^{\theta -1}}{\left[1-{\left(1-exp\left\{-{\left[exp\left(x^{\beta }\right)-1\right]}^{2}\right\}\right)}^{\theta }\right]{\left(1-exp\left\{-{\left[exp\left(x^{\beta }\right)-1\right]}^{2}\right\}\right)}^{1-\theta }},$$

and

$$H_{\theta ,\beta }\left(x\right)=-log\left[1-{\left(1-exp\left\{-{\left[exp\left(x^{\beta }\right)-1\right]}^{2}\right\}\right)}^{\theta }\right],$$

respectively. The PDF and HRF plots of the BXW distribution for some parameters are given below. Hereafter, we provide a very useful linear representation for the BXW density function. 

First, we consider the two power series:

(6) $${\left(1-\frac{u_{1}}{u_{2}}\right)}^{b-1}=\sum_{i=0}^{\infty }\frac{\Gamma \left(b\right)}{i!\Gamma \left(b-i\right)}{\left(-\frac{u_{1}}{u_{2}}\right)}^{i}\;\mathrm{and}\;{\left(1-\frac{u_{1}}{u_{2}}\right)}^{-b}=\sum_{i=0}^{\infty }\frac{\Gamma \left(b+i\right)}{i!\Gamma \left(b\right)}{\left(\frac{u_{1}}{u_{2}}\right)}^{i},$$

where $\left|\frac{u_{1}}{u_{2}}\right|<1$ and b>0. By using the above power series, and after performing some algebra, the PDF (5) can be expressed as:

(7) $$f_{\theta ,\beta }\left(x\right)=\overset{\infty }{\underset{j,k=0}{\sum }}\omega_{j,k}\;\pi_{k+2\left(j+1\right)}\left(x\right),$$

where

$$\omega_{j,k}=2\theta \frac{{\left(-1\right)}^{j}\Gamma \left(\theta \right)\Gamma \left(2j+k+3\right)}{j!k!\left[k+2\left(j+1\right)\right]\Gamma \left(2j+3\right)}\overset{\infty }{\underset{i=0}{\sum }}\frac{{\left(-1\right)}^{i}{\left(i+1\right)}^{j}}{i!\Gamma \left(\theta -i\right)},$$

and

$$\pi_{k+2\left(j+1\right)}\left(x\right)=\left[k+2\left(j+1\right)\right]\beta x^{\beta -1}exp\left(-x^{\beta }\right){\left[1-exp\left(-x^{\beta }\right)\right]}^{k+2j+1}.$$

Equation (7) reveals that the PDF of X can be expressed as a linear mixture of Exp-W densities. Therefore, several mathematical properties of the new family can be obtained by knowing those of the Exp-W distribution. Similarly, the CDF of the BXW distribution can also be expressed as a mixture of Exp-W, CDF given by:

(8) $$F_{\theta ,\beta }\left(x\right)=\overset{\infty }{\underset{j,k=0}{\sum }}\omega_{j,k}\Pi_{k+2\left(j+1\right)}\left(x\right),$$

where

$$\Pi_{k+2\left(j+1\right)}\left(x\right)={\left[1-\exp\left(-x^{\beta }\right)\right]}^{k+2\left(j+1\right)}$$

is the CDF of the Exp-W distribution with power parameter k+2(j+1). According to [25], the BXW distribution can be expressed through the following functional composition:

$$F_{\theta ,\beta }\left(x\right)=G_{2}^{\theta }{}^{\circ}Q_{L}{}^{\circ}G_{\beta }\left(x\right),$$

where $G_{2}^{\theta }$ is $G_{2}^{}$ to the power *θ* and $Q_{L}$ refers to the quantile function of a loglogistic model with parameters equal to 1, namely, the odds function:

$$Q_{L}\left(p\right)=\frac{p}{1-p}$$

Reference [25] also studied the function $Q_{L}\left(F\right),$ where *F* is any given CDF. In particular, ${}^{\circ}Q_{L}G_{\beta }\left(x\right)$ is a convex function; therefore, stochastic ordering properties of the BXW model can be derived straightforwardly through Theorem 1 of [25].

Henceforth, $Y_{k+2\left(j+1\right)}$ denotes a RV with Exp-W distribution by power parameter k+2(j+1). The PDF and CDF of Y are then given by

$$g_{\beta }\left(y\right)=\beta \left[k+2\left(j+1\right)\right]y^{\beta -1}exp\left(-y^{\beta }\right){\left[1-exp\left(-y^{\beta }\right)\right]}^{k+2j+1},$$

and

$$G_{\beta }\left(y\right)={\left[1-exp\left(-y^{\beta }\right)\right]}^{k+2\left(j+1\right)}.$$

For any r>−β, the rth ordinary and incomplete moments of Y are given by

$$\mu_{r}^{\prime }=\overset{\infty }{\underset{h=0}{\sum }}w_{h|\left(r,k+2\left(j+1\right)\right)}\;\Gamma \left(1+\frac{r}{\beta }\right),$$

and

$$\varphi_{r}\left(y\right)=\overset{\infty }{\underset{h=0}{\sum }}w_{h|\left(r,k+2\left(j+1\right)\right)}\;\gamma \left(1+\frac{r}{\beta },y^{\beta }\right),$$

respectively, where

$$w_{h|\left(r,k+2\left(j+1\right)\right)}=\frac{\left[k+2\left(j+1\right)\right]{\left(-1\right)}^{h}\Gamma \left(k+2\left(j+1\right)\right)}{h!\Gamma \left(k+2\left(j+1\right)-h\right){\left(h+1\right)}^{1+\frac{r}{\beta }}}.$$

**Figure 1:**
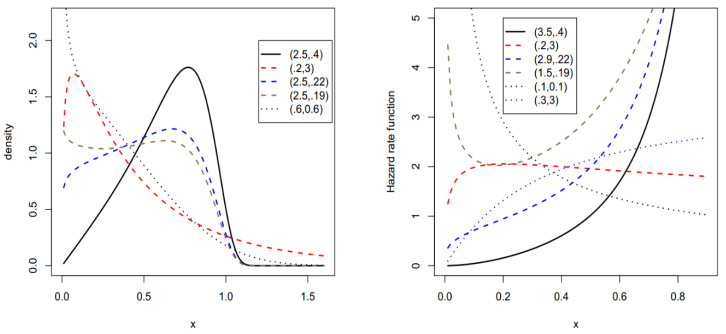
Plots of PDF (**lest panel**) and HRF (**right paned**) of the Burr X Weibull (BXW) model.

Figure 1 below gives some pots of probability density function (PDF) (lest panel) and hazard rate function (HRF) (right paned) of the Burr X Weibull (BXW) model to illustrate the importance of the new model.

## 3. Properties

### 3.1. Some Moments

The rth ordinary moment of X is given by:

$$\mu_{r}^{\prime }=E\left(X^{r}\right)=\int_{-\infty }^{\infty }x^{r}f\left(x\right)dx.$$

We then obtain (for any r>−β)

(9) $$\mu_{r}^{\prime }=\overset{\infty }{\underset{j,k,h=0}{\sum }}w_{h|\left(r,k+2\left(j+1\right)\right)}\omega_{j,k}\Gamma \left(1+\frac{r}{\beta }\right).$$

Setting r = 1 in Equation (9), we have the mean of X. The last integration can be computed numerically for most parent distributions. The sth incomplete moment, say $u_{s}\left(t\right)$, of X can be expressed using Equation (7), for s>−β, as

(10) $$u_{s}\left(t\right)=\int_{-\infty }^{t}x^{s}f\left(x\right)dx=\overset{\infty }{\underset{i,j,k=0}{\sum }}w_{h|\left(r,k+2\left(j+1\right)\right)}\omega_{j,k}\;\gamma \left(1+\frac{s}{\beta },t^{\beta }\right).$$

The mean deviations about the mean $\delta_{1}=E\left(\left|X-\mu_{1}^{\prime }\right|\right)$ and about the median $\delta_{2}=E\left(\left|X-M\right|\right)$ of X are given by $\delta_{1}=2\mu_{1}^{\prime }F\left(\mu_{1}^{\prime }\right)-2u_{1}\left(\mu_{1}^{\prime }\right)$ and $\delta_{2}=\mu_{1}^{\prime }-2u_{1}\left(M\right)$, respectively, where $\mu_{1}^{\prime }=E\left(X\right)$, M is the median of X, $F\left(\mu_{1}^{\prime }\right)$ is easily calculated from Equation (4), and $u_{1}\left(t\right)$ is the first incomplete moment given by (10) with s = 1, as

$$u_{1}\left(t\right)=\overset{\infty }{\underset{i,j,k=0}{\sum }}w_{h|\left(1,k+2\left(j+1\right)\right)}\omega_{j,k}\;\gamma \left(1+\frac{1}{\beta },t^{\beta }\right).$$

The main applications of the first incomplete moment refer to the mean deviations and the Bon-ferroni and Lorenz curves. These curves are very useful in economics, reliability, demography, insurance, and medicine. The Lorenz, say $L_{F}$ and Bonferroni, say B[F(x)], curves are defined by

$$L_{F}\left(x\right)=\frac{1}{E\left(X\right)}\int_{0}^{x}tf\left(t\right)dt$$

and

$$B\left[F\left(x\right)\right]=\frac{1}{E\left(X\right)F\left(x\right)}\int_{0}^{x}tf\left(t\right)dt=\frac{L_{F}\left(x\right)}{F\left(X\right)},$$

respectively. Here, we derive $L_{F}\left(x\right)$ and B[F(x)] curves for the BXW distribution as follows:

$$L_{F}\left(x\right)=\frac{\sum_{i,j,k=0}^{\infty }w_{h|\left(1,k+2\left(j+1\right)\right)}\omega_{j,k}\;\gamma \left(1+\frac{1}{\beta },t^{\beta }\right)}{\sum_{i,j,k=0}^{\infty }w_{h|\left(1,k+2\left(j+1\right)\right)}\omega_{j,k}\Gamma \left(1+\frac{1}{\beta }\right)}$$

and

$$B\left[F\left(x\right)\right]=\frac{\sum_{i,j,k=0}^{\infty }w_{h|\left(1,k+2\left(j+1\right)\right)}\omega_{j,k}\;\gamma \left(1+\frac{1}{\beta },t^{\beta }\right)}{\sum_{i,j,k=0}^{\infty }w_{h|\left(1,k+2\left(j+1\right)\right)}\omega_{j,k}\Gamma \left(1+\frac{1}{\beta }\right)}{\left(1-\exp\left\{-{\left[exp\left(x^{\beta }\right)-1\right]}^{2}\right\}\right)}^{-\theta }.$$

### 3.2. Generating Function

Let $M\left(t\right)=M_{k+2\left(j+1\right)}\left(t\right)$ be the moment generating function (MGF) of $Y_{k+2\left(j+1\right)}$. Therefore, using Equation (7), the MGF of X, say M(t)=E(exp(tx)), is given by

$$M\left(t\right)=\overset{\infty }{\underset{j,k=0}{\sum }}\omega_{j,k}M_{k+2\left(j+1\right)}\left(t;\beta \right),$$

where $M_{k+2\left(j+1\right)}\left(t;\beta \right)$ is the MGF of the Exp-W model with power parameter k+2(j+1).

### 3.3. Probability Weighted Moments (PWMs)

The (s,r)th probability weighted moment (PWM) of X, following the BXW distribution, say $\rho_{s,r}$, is formally defined by:

$$\rho_{s,r}=E\left\{X^{s}F{\left(X\right)}^{r}\right\}=\int_{-\infty }^{\infty }x^{s}F{\left(x\right)}^{r}f\left(x\right)dx.$$

Using Equations (4) and (5), we can write

$$f\left(x\right)F{\left(x\right)}^{r}=\overset{\infty }{\underset{j,k=0}{\sum }}q_{j,k}\beta \left[k+2\left(j+1\right)\right]x^{\beta -1}\exp\left(-x^{\beta }\right){\left[1-\exp\left(-x^{\beta }\right)\right]}^{2j+k+1},$$

where

$$q_{j,k}=\frac{{\left(-1\right)}^{j}\Gamma \left(2j+k+3\right)}{j!k!\Gamma \left(2j+3\right)}\overset{\infty }{\underset{i=0}{\sum }}\frac{2\theta {\left(-1\right)}^{i}{\left(i+1\right)}^{j}\left(\begin{array}{c} \theta \left(r+1\right)-1\\ i \end{array}\right)}{\left[k+2\left(j+1\right)\right]}.$$

Then, the (s,r)th PWM of X can be expressed (for s>−β) as

$$\rho_{s,r}=\overset{\infty }{\underset{j,k=0}{\sum }}q_{j,k}E\left(Y_{2j+k+2}^{s}\right)=\overset{\infty }{\underset{i,j,k=0}{\sum }}w_{i|\left(s,k+2\left(j+1\right)\right)}q_{j,k}\Gamma \left(1+\frac{s}{\beta }\right).$$

### 3.4. Order Statistics

Let $X_{1},X_{2},\ldots ,X_{n}$ be a random sample from the BXW distribution and let $X_{\left(1\right)},X_{\left(2\right)},\ldots ,X_{\left(n\right)}$ be the corresponding order statistics. The PDF of the ith order statistic, say $X_{i:n}$, can be written as

(11) $$f_{i:n}\left(x\right)=\frac{f\left(x\right)}{B\left(i,n+i-1\right)}\overset{n-i}{\underset{j=0}{\sum }}{\left(-1\right)}^{j}\left(\begin{array}{c} n-i\\ j \end{array}\right)F^{j+i-1}\left(x\right),$$

where B(·, ·) is the beta function. Substituting Equations (4) and (5) in Equation (11) and using a power series expansion, we have:

$$f\left(x\right)F{\left(x\right)}^{i+j-1}=\overset{\infty }{\underset{w,k=0}{\sum }}b_{w,k}\beta \left[k+2\left(w+1\right)\right]x^{\beta -1}exp\left(-x^{\beta }\right){\left[1-exp\left(-x^{\beta }\right)\right]}^{2w+k+1}$$

where

$$b_{w,k}=2\theta \frac{{\left(-1\right)}^{w}\Gamma \left(2w+k+3\right)}{w!k!\Gamma \left(2w+3\right)}\overset{\infty }{\underset{m=0}{\sum }}\frac{{\left(-1\right)}^{m}{\left(m+1\right)}^{w}}{k+2\left(w+1\right)}\left(\begin{array}{c} \theta \left(i+j\right)-1\\ m \end{array}\right).$$

The PDF of $X_{i:n}$ can be expressed as:

$$f_{i:n}\left(x\right)=\overset{\infty }{\underset{w,k=0}{\sum }}\overset{n-i}{\underset{j=0}{\sum }}\frac{{\left(-1\right)}^{j}}{B\left(i,n-i+1\right)}\left(\begin{array}{c} n-i\\ j \end{array}\right)b_{w,k}\pi_{k+2\left(w+1\right)}\left(x\right).$$

The density function of the BXW order statistics is a mixture of Exp-W PDF. Based on the last equation, we note that the properties of $X_{i:n}$ follow from those properties of $Y_{2w+k+2}$. For example, the moments of $X_{i:n}$ can be given (for q>−β) by:

(12) $$E\left(X_{i:n}^{q}\right)=\overset{\infty }{\underset{w,k,h=0}{\sum }}\overset{n-i}{\underset{j=0}{\sum }}\frac{{\left(-1\right)}^{j}\left(\begin{array}{c} n-i\\ j \end{array}\right)}{B\left(i,n-i+1\right)}w_{h|\left(q,k+2\left(w+1\right)\right)}b_{w,k}\Gamma \left(1+\frac{q}{\beta }\right).$$

### 3.5. Renyi and δ −Entropies

The Renyi entropy of a RV X represents a measure of variation of the uncertainty. The Renyi entropy is defined by:

$$I_{\delta }\left(X\right)=\frac{1}{1-\delta }log\int_{-\infty }^{\infty }f{\left(x\right)}^{\delta }dx{|}_{\left(\delta >0,\;\delta \neq 1\right)}.$$

Using the PDF (5), the last equation the Renyi entropy of X is given by

$$I_{\delta }\left(X\right)=\frac{1}{1-\delta }log\left[\overset{\infty }{\underset{i,j,k,h=0}{\sum }}t_{i,j,k,h}\Gamma \left(1+\frac{\delta \beta -\delta -\beta +1}{\beta }\right)\right],$$

where

$$t_{i,j,k,h}=\frac{2^{\delta }\theta^{\delta }\beta^{\delta }{\left(-1\right)}^{i+j+h}\Gamma \left(3\delta +2j+k\right)\left(\begin{array}{c} \theta \delta -\delta \\ i \end{array}\right)\left(\begin{array}{c} \delta +2j+k\\ h \end{array}\right)}{j!k!{\left(\delta +i\right)}^{-j}\Gamma \left(3\delta +2j\right){\left(\delta +h\right)}^{\frac{\delta \left(\beta -1\right)+1}{\beta }}}.$$

The δ-entropy, say $H_{\delta }\left(X\right)$, can be obtained (for, δ>0, δ≠1) as:

$$H_{\delta }\left(X\right)=\frac{1}{1-\delta }log\left\{1-\left[\overset{\infty }{\underset{i,j,k,h=0}{\sum }}t_{i,j,k,h}\Gamma \left(1+\frac{\delta \left(\beta -1\right)-\beta +1}{\beta }\right)\right]\right\}.$$

## 4. Classical Parameter Estimation

Several approaches for parameter estimation were proposed in the literature. In this article, we will consider the following methods:

**I.**  The maximum likelihood method;
**II.**  Method of Cramer-Von-Mises estimation;
**III.**  Method of percentile estimation;
**IV.**  Method of L-moments.

### 4.1. The Maximum Likelihood Method

Let $x_{1},x_{2},\ldots ,x_{n}$ be a random sample from the BXW distribution with parameters θ and β. Let $\phi ={\left(\theta ,\beta \right)}^{T}$ be the 2 × 1 parameter vector. For determining the MLE of ϕ, we have the log-likelihood function:

$$\begin{array}{c} l=l\left(\phi \right)=nlog2+nlog\theta +nlog\beta +\left(\beta -1\right)\overset{n}{\underset{i=1}{\sum }}\log\left(x_{i}\right)+\overset{n}{\underset{i=1}{\sum }}log\left(1-s_{i}\right)\\ +\overset{n}{\underset{i=1}{\sum }}log\left(2x_{i}^{\beta }-z_{i}^{2}\right)+\left(\theta -1\right)\overset{n}{\underset{i=1}{\sum }}log\left(1-exp\left(-z_{i}^{2}\right)\right), \end{array}$$

where

$$z_{i}=\frac{1-s_{i}}{s_{i}},$$

and

$$s_{i}=\exp\left(-x_{i}^{\beta }\right).$$

The components of the score vector

$$U\left(\phi \right)=\frac{dl}{d\phi }={\left(\frac{dl\left(\phi \right)}{d\theta },\frac{dl\left(\phi \right)}{d\beta }\right)}^{T}$$

are

$$U\left(\theta \right)=\frac{n}{\theta }+\overset{n}{\underset{i=1}{\sum }}log\left(1-exp\left(-z_{i}^{2}\right)\right),$$

and

$$\begin{array}{c} U\left(\beta \right)=\frac{n}{\beta }+\overset{n}{\underset{i=1}{\sum }}log\left(x_{i}\right)-\overset{n}{\underset{i=1}{\sum }}\frac{b_{i}}{1-s_{i}}+\overset{n}{\underset{i=1}{\sum }}\frac{2x_{i}^{\beta }log\left(x_{i}\right)-2m_{i}z_{i}}{2x_{i}^{\beta }-z_{i}^{2}}\\ +\left(\theta -1\right)\overset{n}{\underset{i=1}{\sum }}\frac{2m_{i}z_{i}exp\left(-z_{i}^{2}\right)}{1-exp\left(-z_{i}^{2}\right)}, \end{array}$$

where

$$b_{i}=-x_{i}^{\beta }exp\left(-x_{i}^{\beta }\right)log\left(x_{i}\right),$$

and

$$m_{i}=\frac{b_{i}}{s_{i}^{2}}.$$

Setting the nonlinear system of equations U(θ)=0 and U(β)=0 and solving them simultaneously yields the $\hat{\phi }={\left(\hat{\theta },\hat{\beta }\right)}^{T}$. To solve these equations, it is usually more convenient to use nonlinear optimization methods, such as the quasi-Newton algorithm to numerically maximize l(ϕ). 

### 4.2. Cramer-Von-Mises Estimation Method

The Cramer-Von-Mises estimation method of the parameters is based on the theory of minimum distance estimation (see [26]). The Crammer-Von-Mises estimates (CVMEs) of the parameter θ and β are obtained by minimizing the following expression, with respect to (w.r.t.), the parameters θ and β, respectively:

$$CVM_{\left(\phi \right)}=\frac{1}{12n}+\overset{n}{\underset{i=1}{\sum }}{\left[F_{\theta ,\beta }\left(x_{i}\right)-\frac{-1+2i}{2n}\right]}^{2},$$

then

$$CVM_{\left(\phi \right)}=\overset{n}{\underset{i=1}{\sum }}{\left({\left\{1-exp\left[-{\left[exp\left(x_{i}^{\beta }\right)-1\right]}^{2}\right]\right\}}^{\theta }-\frac{-1+2i}{2n}\right)}^{2}.$$

The Cramer-Von-Mises estimates (CVMEs) of the parameters are obtained by solving the following non-linear equations:

$$\overset{n}{\underset{i=1}{\sum }}\left({\left\{1-exp\left[-{\left[exp\left(x_{i}^{\beta }\right)-1\right]}^{2}\right]\right\}}^{\theta }-\frac{-1+2i}{2n}\right)\eta_{\left(\theta \right)}\left(x_{i},\phi \right)=0,$$

$$\overset{n}{\underset{i=1}{\sum }}\left({\left\{1-exp\left[-{\left[exp\left(x_{i}^{\beta }\right)-1\right]}^{2}\right]\right\}}^{\theta }-\frac{-1+2i}{2n}\right)\eta_{\left(\beta \right)}\left(x_{i},\phi \right)=0,$$

where $\eta_{\left(\theta \right)}\left(x_{i},\theta ,\beta \right)$ and $\eta_{\left(\beta \right)}\left(x_{i},\theta ,\beta \right)$ are the values of the first derivatives of the CDF of BXW distribution w.r.t. θ,β, respectively.

### 4.3. Method of Percentile Estimation

Let ${ϒ}_{\left(i\right)}=\frac{i}{1+n}$ be an estimate of $F_{\left(\phi \right)}\left(x_{\left(i\right)}\right)$, then the percentile estimators (PerEs) of θ and β can be obtained by minimizing the function

$$\overset{n}{\underset{i=1}{\sum }}{\left(x_{\left(i\right)}-{\left\{log\left[1+\sqrt{-log\left(1-{ϒ}_{\left(i\right)}^{\frac{1}{\theta }}\right)}\right]\right\}}^{\frac{1}{\beta }}\right)}^{2},$$

with respect to θ and β. Additionally, the PerEs can be obtained by solving the following nonlinear equations:

$$0=\overset{n}{\underset{i=1}{\sum }}\left(x_{\left(i\right)}-{\left\{log\left[1+\sqrt{-log\left(1-{ϒ}_{\left(i\right)}^{\frac{1}{\theta }}\right)}\right]\right\}}^{\frac{1}{\beta }}\right)\tau_{\left(\phi \right)}^{\left(1\right)}\left(x_{\left(i\right)}\right),$$

$$0=\overset{n}{\underset{i=1}{\sum }}\left(x_{\left(i\right)}-{\left\{log\left[1+\sqrt{-log\left(1-{ϒ}_{\left(i\right)}^{\frac{1}{\theta }}\right)}\right]\right\}}^{\frac{1}{\beta }}\right)\tau_{\left(\phi \right)}^{\left(2\right)}\left(x_{\left(i\right)}\right),$$

where

$$\tau_{\left(\phi \right)}^{\left(1\right)}\left(x_{\left(i\right)}\right)=\frac{\partial }{\partial \theta }\left(x_{\left(i\right)}-{\left\{log\left[1+\sqrt{-log\left(1-{ϒ}_{\left(i\right)}^{\frac{1}{\theta }}\right)}\right]\right\}}^{\frac{1}{\beta }}\right)$$

and

$$\tau_{\left(\phi \right)}^{\left(2\right)}\left(x_{\left(i\right)}\right)=\frac{\partial }{\partial \beta }\left(x_{\left(i\right)}-{\left\{log\left[1+\sqrt{-log\left(1-{ϒ}_{\left(i\right)}^{\frac{1}{\theta }}\right)}\right]\right\}}^{\frac{1}{\beta }}\right).$$

### 4.4. Method of L-Moments

The L-moments are analogous to the ordinary moments but can be estimated by linear combinations of order statistics. They exist whenever the mean of the distribution exists, even though some higher moments may not exist and are relatively robust to the effects of outliers. Based upon the moments of the order statistics, we can derive explicit expressions for the L-moments of X as infinite weighted linear combinations of the means of suitable BXW order statistics. The L-moments for the population can be obtained from:

$$\lambda_{\left(r\right)}=\frac{1}{r}\overset{r-1}{\underset{d=0}{\sum }}{\left(-1\right)}^{d}\left(\begin{array}{c} r-1\\ d \end{array}\right)E\left(X_{\left(r-d\;:\;d\right)}\right){|}_{\left(r\geq 1\right)}.$$

The first four L-moments are given by

$$\lambda_{\left(1\right)}\left(\phi \right)=E\left(X_{\left(1:1\right)}\right)=L\left(1\right)$$

$$\lambda_{\left(2\right)}\left(\phi \right)=\frac{1}{2}E\left(X_{\left(2:2\right)}-X_{\left(1:2\right)}\right)=\frac{1}{2}\left(\mu_{2:2}^{\prime }-\mu_{1:2}^{\prime }\right)=L\left(2\right),$$

where $L\left(i\right){|}_{\left(i=1,2\right)}$ is the L-moments for the sample. The L-moment estimators of the parameters θ and  β can then be obtained numerically.

## 5. Simulation Studies

### 5.1. Simulation Study for Assessing the Maximum Likilihood Method 

#### 5.1.1. Numerical Assessment

In this section, we study the performance and accuracy of maximum likelihood estimates of the BXW model parameters by conducting various simulations for different sample sizes and different parameter values. The method for generating samples from the BXW distribution is performed by inverse CDF of BXW and uniform RV, as follows:

If

(13) $$x={\left\{log\left[1+\sqrt{-log\left(1-u^{\frac{1}{\theta }}\right)}\right]\right\}}^{\frac{1}{\beta }},$$

where u~U(0,1), then X~BXW(ϕ). The simulation study is repeated for N = 5000 times, each with sample size n = 100, 200, and 500 and parameter values (θ,β)=(0.4,2.5), (3,0.02), (0.6,0.6) and (0.19,2.5). Two quantities are computed in this simulation study:

1. Average bias of the MLE $\hat{\varepsilon }$ of the parameter ε=θ,β:

$$Bias_{\varepsilon }\left(n\right)=\frac{1}{5000}\overset{5000}{\underset{i=1}{\sum }}\left(\hat{\varepsilon_{i}}-\varepsilon \right).$$

2. Mean square error (MSE) of the MLE $\hat{\varepsilon }$ of the parameter ε=θ,β:

$$MSE_{\varepsilon }\left(n\right)=\frac{1}{5000}\overset{5000}{\underset{i=1}{\sum }}{\left(\hat{\varepsilon_{i}}-\varepsilon \right)}^{2}.$$

Table 1 presents the Bias and mean square error (MSE) values of the parameters θ and β for different sample sizes. From the results, we can verify that, as the sample size n increases, the MSEs decay toward zero. We also observe that for all the parametric values, the biases decrease as the sample size n increases.

#### 5.1.2. Graphical Assessment

Graphically, we could perform the simulation experiments to assess the finite sample behavior of the MLEs. The assessment was based on the following algorithm:

1. Use Equation (13) to generate 1000 samples of size n from the BXW distribution;
2. Compute the MLEs for the 1000 samples;
3. Compute the standard errors (SEs) of the MLEs for the 1000 samples (the standard errors (SEs) were computed by inverting the observed information matrix).
4. Compute the biases and mean square errors given for θ,β.

**Figure 2:**
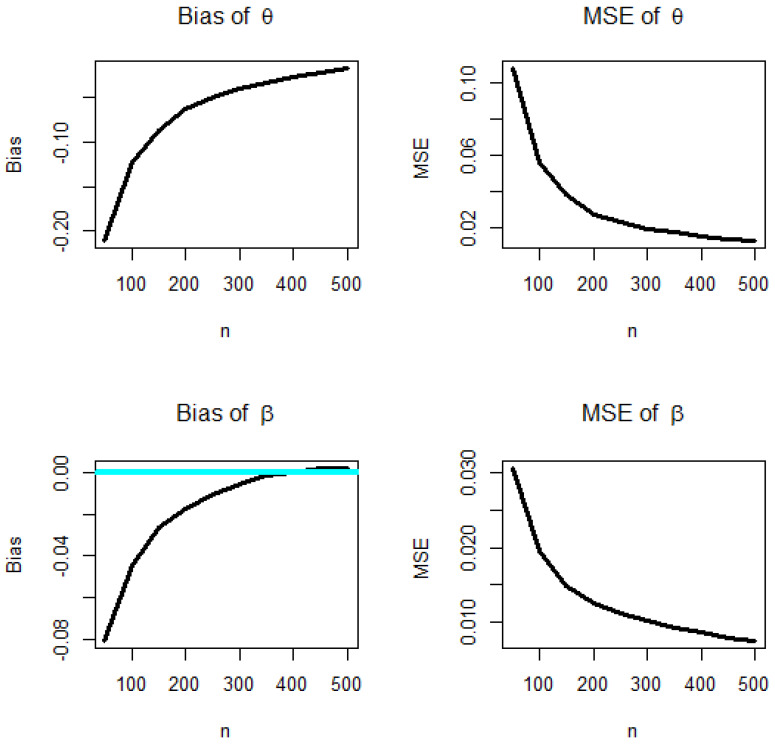
Biases (**left panels**) and MSEs (**right panels**) for θ,β, and n = 50, 100, …, 1000 for the BXW model.

We repeated these steps for n = 50, 100, …, 1000 with θ=1, β=1 computing biases and MSEs. Figure 2 (left panel) shows how the two biases vary, with respect to n. Figure 2 (right panel) shows how the two MSEs vary, with respect to n. The broken lines in Figure 2 correspond to the biases at 0. From Figure 2, the biases for each parameter are generally negative and decrease to zero as n→∞, the MSEs for each parameter decrease to zero as n→∞.

### 5.2. Simulation Studies for Comparing Non-Bayesian Estimation Methods

A Markov chain Monte-Carlo (MCMC) simulation study was performed for this section to assess and compare the performance of the different estimators of the unknown parameters of the new distribution. This performance was assessed using the average values (AVs) of estimates and the mean square errors (MSEs). First, we generated 1000 samples of the BXW distribution, where n = (20, 50, 150, 300), and chose:
**Parameters****I****II****III**θ20.66β0.50.40.1

The AVs and MSEs of MLEs, PerEs, and L-Moments were obtained and listed in Table 2, Table 3, Table 4 and Table 5. From Table 2, Table 3, Table 4 and Table 5, we noted that all methods performed well.

## 6. Non-Bayesian Uncensored Applications

### 6.1. Non-Bayesian Uncensored Applications for Comparing Models

We provided two applications with two real data sets to prove the flexibility of the BXW distribution. We fitted some of the well-known two parameter lifetime distributions, such as the Weibull, gamma, generalized exponential (GE) ([27]), exponential geometric (EG) ([28]), exponential Poisson (EP) ([29]), and complementary exponential geometric (CEG) ([30]) distributions, into two real data sets.

The first data set was given by [31] on the prices of the 31 different children’s wooden toys on sale in a Suffolk craft shop in April 1991. The data set consisted of 31 observations. The second data set was an uncensored data set from [32], consisting of 34 observations on vinyl chloride data obtained from clean upgradient monitoring wells in mg/L. These data were also analyzed by [33]. The MLE of parameters, such as the maximized log-likelihood function, Akaike information criterion (AIC), Bayesian information criterion (BIC), Hannan-Quinn information criterion (HQIC), consistent Akaike information criterion (CAIC) ([30]), Anderson-Darling ($A^{*}$), and Cramer-von-Mises ($W^{*}$) statistics, were determined by fitting the two parameter distributions using the two data sets. The statistics $A^{*}$ and $W^{*}$ were described by [34]. In general, the smaller values of these statistics showed the better fit to the data sets. The MLEs were computed using the limited-memory quasi-Newton code for bound-constrained optimization (L-BFGS-B). The estimated parameters, shown in Table 6 and Table 7, were based on MLE procedure reports, whereas the values of goodness-of-fit statistics are given in Table 8 and Table 9. In the applications, the information about the hazard shape helped in selecting a particular model. For this aim, a device called the total time on test (TTT) plot ([35]) was useful. 

It is convex shape for decreasing hazards and a concave shape for increasing hazards.

**Figure 3:**
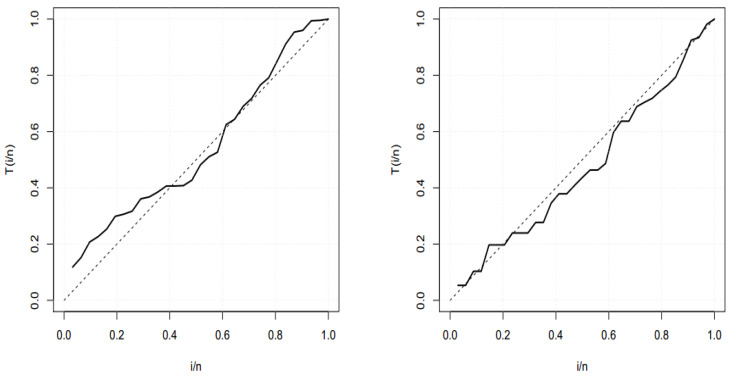
Total time on test (TTT) plot for the first dataset (**left figure**) and for the second dataset (**right figure**).

**Figure 4:**
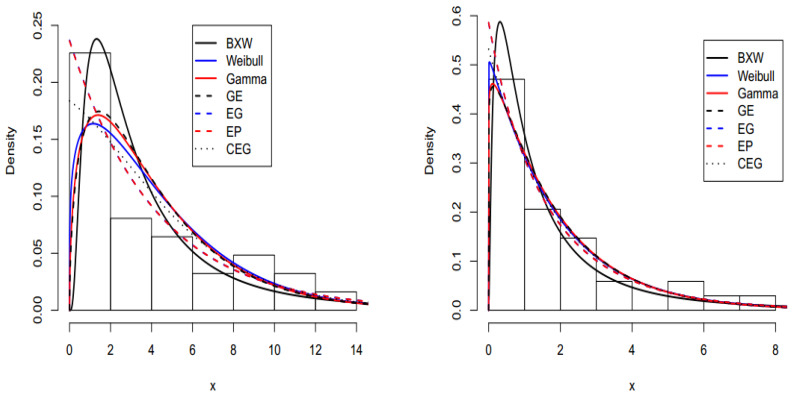
TTT plot for the first dataset (**left figure**) and for the second dataset (**right figure**).

**Figure 5:**
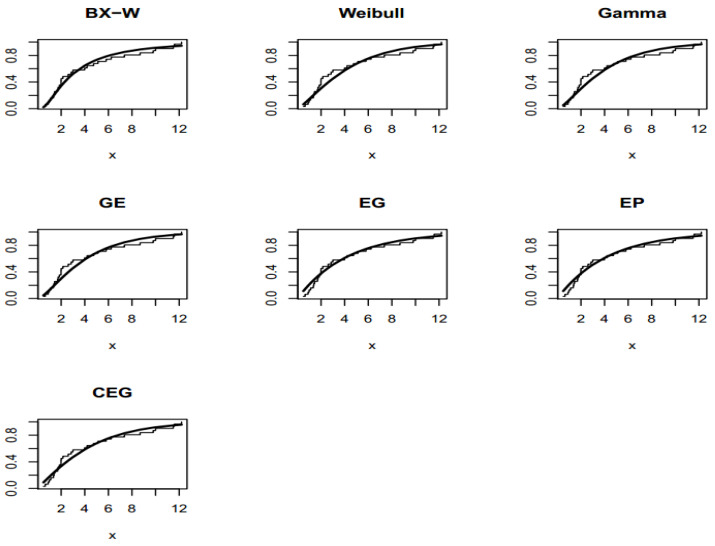
Fitted cumulative distribution functions (CDFs) on the empirical CDF of the first data set.

**Figure 6:**
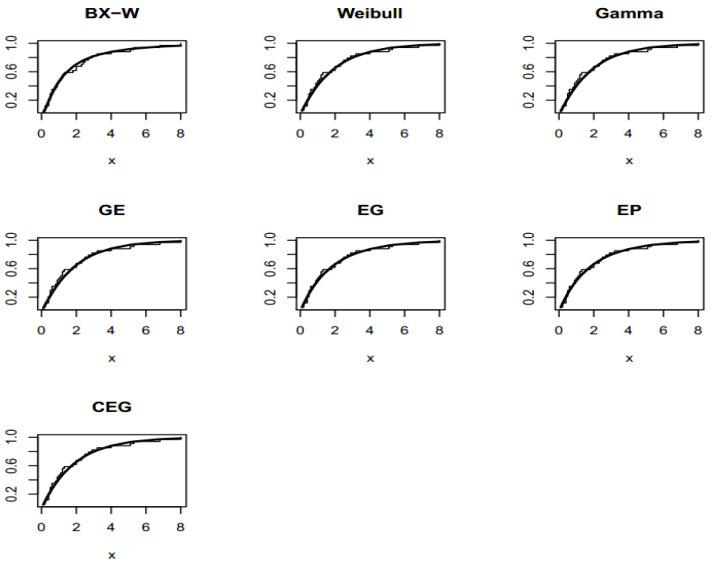
Fitted CDFs on the empirical CDF of the second data set.

The TTT plot for both data sets are presented in Figure 3. These figures indicate that the first and second datasets have bathtub and constant failure rate functions. In both real data sets, the results show that the BXW distribution yields a better fit than other distributions. These conclusions are also confirmed by Figure 4, Figure 5 and Figure 6.

### 6.2. Uncensored Applications for Comparing the Non-Bayesian Methods

Table 10 and Table 11 give the values of estimators, along with the $W^{*}$ and $A^{*}$ statistics for all methods. From Table 10, we conclude that the PerEs method was the best method for the first data; however, all other methods performed well. From Table 11. we conclude that the L-moment method was the best method for the second data; however, all other methods performed well.

## 7. Censored Maximum Likelihood Estimation

Suppose that $X_{1},X_{2},\ldots \ldots ,X_{n}$ is a random sample with right censoring from the BXW distribution. The observed data $x_{i}=min(X_{i},C_{i});$ i=1,2,…,n are the minimum of the survival time $X_{i}$ and censoring time $C_{i}$ for each subject in the sample. Therefore, $x_{i}$ can be written in the form ${\left(x_{i},\delta_{i}\right)}_{i=1,\ldots ,n}$, where $\delta_{i}=1$ if $X_{i}$ is the moment of failure (complete observation) and $\delta_{i}=0$ if $X_{i}$ is the moment of censoring. The right censoring was assumed to be non-informative, so the expression of the likelihood function is:

$$l_{\phi }\left(x\right)=\Pi_{i=1}^{n}f_{\phi }{\left(x_{i}\right)}^{\delta_{i}}R_{\phi }{\left(x_{i}\right)}^{1-\delta_{i}}{|}_{\delta_{i}=1_{X_{i}<C_{i}}},$$

where $R_{\phi }\left(x_{i}\right)$ refers to the RF. The log-likelihood function of the BXW distribution is:

$$L\left(\phi \right)=\overset{n}{\underset{i=1}{\sum }}\delta_{i}lnf\left(x_{i},\varphi \right)+\overset{n}{\underset{i=1}{\sum }}\left(1-\delta_{i}\right)lnR_{\phi }\left(x_{i}\right)$$

$$=\overset{n}{\underset{i=1}{\sum }}\delta_{i}\left[\begin{array}{c} ln(2\theta \beta )+\left(\beta -1\right)ln(x_{i})+2x_{i}^{\beta }-{\left(e^{x_{i}^{\beta }}-1\right)}^{2}\\ -\left(1-\theta \right)ln\left(1-exp\left\{-{\left[e^{x^{\beta }}-1\right]}^{2}\right\}\right) \end{array}\right]$$

$$+\overset{n}{\underset{i=1}{\sum }}\left(1-\delta_{i}\right)ln\left[1-{\left(1-exp\left\{-{\left[e^{x^{\beta }}-1\right]}^{2}\right\}\right)}^{\theta }\right]$$

and the score functions are obtained as follows:

$$\frac{\partial L\left(\phi \right)}{\partial \theta }=\overset{n}{\underset{i=1}{\sum }}\delta_{i}\left[\frac{1}{\theta }+ln(1-e^{-z_{i}^{2}})\right]-\overset{n}{\underset{i=1}{\sum }}\left(1-\delta_{i}\right)\frac{\left(1-e^{-z_{i}^{2}}{)}^{\theta }ln(1-e^{-z_{i}^{2}}\right)}{1-{\left(1-e^{-z_{i}^{2}}\right)}^{\theta }}$$

$$\begin{array}{cc} \frac{\partial L\left(\phi \right)}{\partial \beta }=\overset{n}{\underset{i=1}{\sum }}\delta_{i} & \left[\begin{array}{c} \frac{1}{\beta }+\frac{s_{i}x_{i}^{\beta }lnx_{i}}{1-s_{i}}-\frac{2\left(1-\theta \right)z_{i}x_{i}^{\beta }ln(x_{i})e^{x_{i}^{\beta }-z_{i}^{2}}}{1-e^{-z_{i}^{2}}}\\ +ln(x_{i})+2x_{i}^{\beta }ln(x_{i})\left(1-z_{i}e^{x^{\beta }}\right) \end{array}\right]\\ & -\overset{n}{\underset{i=1}{\sum }}\left(1-\delta_{i}\right)\frac{2\theta z_{i}x_{i}^{\beta }ln(x_{i})e^{x_{i}^{\beta }-z_{i}^{2}}{\left(2x_{i}^{\beta }-z_{i}^{2}\right)}^{\theta -1}}{1-{\left(1-e^{-z_{i}^{2}}\right)}^{\theta }}, \end{array}$$

with

$$z_{i}=e^{x_{i}^{\beta }}-1,\;s_{i}=e^{-x_{i}^{\beta }}$$

Maximum likelihood estimators of the unknown parameters can be obtained using various techniques, such as software R, the Expectation–maximization (EM) algorithm, or the Newton–Raphson method.

## 8. Modified Chi-Squared Type Test for Right Censored Data

Methods for testing the validity of parametric models are in constant development, but the presence of censorship make them unavailable. [36,37] proposed a modified chi-squared test based on Kaplan–Meyer estimators. [38] considered modifications of the Kolmogorov–Smirnov statistic, Anderson–Darling statistic, and the Cramer-Von-Mises statistic for accelerate failure models. In this work, we are interested in the modified chi-squared type test, proposed by [39,40,41] for parametric models with right censored data. Based on maximum likelihood estimators on non-grouped data, this statistic test is also based on the differences between the numbers of observed failures and the numbers of expected failures in the grouped intervals chosen. For this, random grouping intervals are considered as data functions. The description of the construction of this chi-squared type test was developed by [42]. The statistic test was defined as follows. Suppose that $X_{1},X_{2},\ldots ,X_{n}$ is a random sample with right censoring from a parametric model and a finite time τ. The statistic test is defined as follows:

$$Y_{n}^{2}=\overset{n}{\underset{j=1}{\sum }}\frac{{\left(U_{j}-e_{j}\right)}^{2}}{U_{j}}+Q$$

where $U_{j}$ and $e_{j}$ are the observed and expected numbers of failure in grouping intervals and Q is:

$$Q=W^{T}{\hat{G}}^{-}W,$$

$$\hat{W}=\hat{C}{\hat{A}}^{-1}Z={\left({\hat{W}}_{1},\ldots ,{\hat{W}}_{s}\right)}^{T},\;$$

$$Z_{j}=\frac{1}{\sqrt{n}}\left(U_{j}-e_{j}\right)$$

$$W_{l}=\overset{r}{\underset{j=1}{\sum }}{\hat{C}}_{lj}{\hat{A}}_{j}^{-1}Z_{j},$$

$$\hat{G}={\left[{\hat{g}}_{ll^{\prime }}\right]}_{s\times s},$$

$${\hat{g}}_{ll^{\prime }}={\hat{ı}}_{ll^{\prime }}-\overset{r}{\underset{j=1}{\sum }}{\hat{C}}_{lj}{\hat{C}}_{l^{\prime }j}{\hat{A}}_{j}^{-1},\;i=1,\ldots ,n,\;j=1,\ldots ,r,\;l,l^{\prime }\;=1,\ldots ,s$$

The limits $a_{j}$ of r random gouging intervals $I_{j}=\left[a_{j-1},a_{j}\right]$ were chosen, such as the expected failure times to fall into these intervals, which were the same for each; j=1,…,r−1, ${\hat{a}}_{r}=max\left(x_{\left(l\right)},\tau \right).$ The estimated ${\hat{a}}_{j}$ is defined by

$${\hat{a}}_{j}=H^{-1}\left(\frac{E_{j}-\sum_{l=1}^{i-1}H_{\hat{\phi }}\left(x_{l}\right)}{n-i+1},\hat{\phi }\right),\;{\hat{a}}_{r}=\overset{\underset{}{}}{max}\left(x_{\left(n\right),}\tau \right)$$

where $H_{\hat{\phi }}\left(x_{l}\right)$ is the cumulative HRF (CHRF) of the model distribution. This statistic test $Y_{n}^{2}$ follows a chi-squared distribution.

### 8.1. Choice of Random Grouping Intervals

Suppose that $X_{1},X_{2},\ldots \ldots ,X_{n}$ is a random sample with right censoring from the BXW distribution and a finite time τ. In our case, the estimated ${\hat{a}}_{j}$ is obtained as follows:

$${\hat{a}}_{j}={\left[ln\left(1\pm \sqrt{-ln\left(1-{\left[1-exp\left\{\frac{\sum_{l=1}^{i-1}H_{\hat{\phi }}\left(x_{l}\right)-E_{j}}{n-i+1}\right\}\right]}^{1/\theta }\right)}\right)\right]}^{1/\beta }$$

where $\hat{\phi }={\left(\hat{\theta },\hat{\beta }\right)}^{T}$ is the maximum likelihood estimator of the unknown parameters $\varphi ={\left(\theta ,\beta \right)}^{T}$ on initial data and $H_{\hat{\phi }}\left(x_{l}\right)$ is the cumulative hazard rate function of the BXW distribution.

### 8.2. Quadratic Form Q

To calculate the quadratic form Q of the statistic $Y_{n}^{2}$, and, as its distribution doesn’t depend on the parameters, we can use the estimated matrices $\hat{W}$ and $\hat{C}$ and the estimated information matrix $\hat{I}.$ The elements of $\hat{C}$ defined by

$${\hat{C}}_{lj}=\frac{1}{n}\overset{n}{\underset{i\;:\;x_{i}\in I_{j}}{\sum }}\delta_{i}\frac{\partial }{\partial {\hat{\phi }}_{l}}lnh_{\hat{\varphi }}\left(x_{i}\right)$$

are obtained as below

$${\hat{C}}_{1j}=\frac{1}{n}\overset{n}{\underset{i\;:\;x_{i}\in I_{j}}{\sum }}\delta_{i}\left[\frac{1}{\theta }+\frac{ln(1-e^{-z_{i}^{2}})}{1-{\left(1-e^{-z_{i}^{2}}\right)}^{\theta }}\right]$$

$${\hat{C}}_{2j}=\frac{1}{n}\overset{n}{\underset{i\;:\;x_{i}\in I_{j}}{\sum }}\delta_{i}\left[\begin{array}{c} \frac{1}{\beta }+ln(x_{i})+\frac{s_{i}x_{i}^{\beta }lnx_{i}}{1-s_{i}}+\frac{2\theta z_{i}x_{i}^{\beta }ln(x_{i})e^{x_{i}^{\beta }-z_{i}^{2}}{\left(2x_{i}^{\beta }-z_{i}^{2}\right)}^{\theta -1}}{1-{\left(1-e^{-z_{i}^{2}}\right)}^{\theta }}\\ -\frac{2\left(1-\theta \right)z_{i}x_{i}^{\beta }ln(x_{i})e^{x_{i}^{\beta }-z_{i}^{2}}}{1-e^{-z_{i}^{2}}}+2x_{i}^{\beta }ln(x_{i})\left(1-z_{i}e^{x^{\beta }}\right) \end{array}\right]$$

Therefore, the estimated matrix $\hat{W}$ can be deducted from $\hat{C}$.

### 8.3. Estimated Information Matrix $\hat{I}$

We need also the information matrix $\hat{I}$ of the BXW distribution with right censoring. After difficult calculations and some simplifications, we obtained the elements of the matrix, as follows:

$${\hat{ı}}_{11}=\frac{1}{n}\overset{n}{\underset{i=1}{\sum }}\delta_{i}{\left(\frac{1}{\theta }+\frac{ln(1-e^{-z_{i}^{2}})}{1-{\left(1-e^{-z_{i}^{2}}\right)}^{\theta }}\right)}^{2}$$

$${\hat{ı}}_{22}=\frac{1}{n}\overset{n}{\underset{i=1}{\sum }}\delta_{i}\left(\begin{array}{c} \frac{1}{\beta }+ln(x_{i})+\frac{s_{i}x_{i}^{\beta }lnx_{i}}{1-s_{i}}+\frac{2\theta z_{i}x_{i}^{\beta }ln(x_{i})e^{x_{i}^{\beta }-z_{i}^{2}}{\left(2x_{i}^{\beta }-z_{i}^{2}\right)}^{\theta -1}}{1-{\left(1-e^{-z_{i}^{2}}\right)}^{\theta }}\\ -\frac{2\left(1-\theta \right)z_{i}x_{i}^{\beta }ln(x_{i})e^{x_{i}^{\beta }-z_{i}^{2}}}{1-e^{-z_{i}^{2}}}+2x_{i}^{\beta }ln(x_{i})\left(1-z_{i}e^{x^{\beta }}\right) \end{array}\right){}^{2}$$

$$\begin{array}{c} {\hat{ı}}_{12}=\frac{1}{n}\overset{n}{\underset{i=1}{\sum }}\delta_{i}\left(\frac{1}{\theta }+\frac{ln(1-e^{-z_{i}^{2}})}{1-{\left(1-e^{-z_{i}^{2}}\right)}^{\theta }}\right)\\ \times \left(\begin{array}{c} \frac{1}{\beta }+ln(x_{i})+\frac{s_{i}x_{i}^{\beta }lnx_{i}}{1-s_{i}}+\frac{2\theta z_{i}x_{i}^{\beta }ln(x_{i})e^{x_{i}^{\beta }-z_{i}^{2}}{\left(2x_{i}^{\beta }-z_{i}^{2}\right)}^{\theta -1}}{1-{\left(1-e^{-z_{i}^{2}}\right)}^{\theta }}\\ -\frac{2\left(1-\theta \right)z_{i}x_{i}^{\beta }ln(x_{i})e^{x_{i}^{\beta }-z_{i}^{2}}}{1-e^{-z_{i}^{2}}}+2x_{i}^{\beta }ln(x_{i})\left(1-z_{i}e^{x^{\beta }}\right) \end{array}\right) \end{array}$$

As all the components of the statistic were given explicitly, we then obtained the statistic test for the BXW distribution with unknown parameters and right censored data. This statistic follows a chi-squared distribution with r degrees of freedom:

$$Y_{n}^{2}\left(\hat{\phi }\right)=\sum_{j=1}^{r}\frac{1}{U_{j}}{\left(U_{j}-e_{j}\right)}^{2}+{\hat{W}}^{T}{\left[{\hat{ı}}_{ll^{\prime }}-\sum_{j=1}^{r}{\hat{C}}_{lj}{\hat{C}}_{l^{\prime }j}{\hat{A}}_{j}^{-1}\right]}^{-1}\hat{W}.$$

## 9. Simulations

An important simulation study is carried out to show the performance of the techniques used and the feasibility of the goodness-of-fit test developed in this work. To this end, we generated N=10,000 right censored samples with different sizes, $n_{1}=20$ and $n_{2}=50,n_{3}=150,n_{4}=300,$ from the BXW model with different parameters. Firstly, we computed the MLEs of the unknown parameters, then the criteria $Y^{2}$ of the corresponding samples were provided.

### 9.1. Censored Maximum Likelihood Estimation for BXW

Using R statistical software and the Barzilai–Borwein (BB) algorithm (see [43]), we calculated the maximum likelihood estimators of the unknown parameters, the corresponding bias, and mean square errors (MSEs). The results are given in Table 12. 

### 9.2. Test Statistic $Y^{2}$

For testing the null hypothesis $H_{0}$, that right censored data come from the BXW model, we computed the criteria statistic $Y_{n}^{2}\left(\phi \right)$, as defined above, for 10,000 simulated samples from the hypothesized distribution with different sizes (n=20,50,150,300). We then calculated empirical levels of significance, when $Y2>\chi_{\varepsilon }^{2}\left(r\right)$, correponding to theoretical levels of significance (ε=0.10,0.05,0.01). We chose r=5. The results are reported in Table 13.

The null hypothesis $H_{0}$, for which simulated samples were fitted by BXW distribution, is widely validated for the different levels of significance. Therefore, the test proposed in this work can be used to fit data from this new distribution.

## 10. Data Analysis

[44] gave data from a laboratory investigation and the number of T days until onset of carcinoma was recorded. The data below concerns a group of 19 rats (Group 1 in Pike’s article). The two observations with asterisks are censorship times, where the data are: 143,164,188, 188, 190, 192, 206, 209, 213, 216, 216 *, 220, 227, 230, 234, 244 *, 246, 265, and 304 (* indicates the censorship). We used the statistics test provided above to verify if these data were modeled by BXW distribution, and, to that end, we first calculated the maximum likelihood estimators of the unknown parameters:

$$\hat{\varphi }={\left(\hat{\theta },\hat{\beta }\right)}^{T}={\left(1.536,0.063\right)}^{T}.$$

Data were grouped into r=4 intervals, $I_{j}$. We give the necessary calculus in Table 14.

We then obtained the value of the statistic test $Y_{n}^{2}$:

$$Y_{n}^{2}=X^{2}+Q=4.106+3.5396=7.6456$$

For significance level ε=0.05, the critical value $\chi_{4}^{2}=9,4877$ was superior than the value of $Y_{n}^{2}=7.6456,$ so we can say that the proposed BXW model fit these data. 

## 11. Conclusions

In this paper, we proposed a new two-parameter Weibull (W) lifetime model based on the Burr X-G (BX-G) class, called the Burr X Weibull (BXW) distribution, which extends the well-known W model. An obvious reason for generalizing a standard distribution is the fact that the generated model can provide more flexibility to analyze real-life data. We provided some of its mathematical and statistical properties. The BXW density function can be expressed as a linear mixture of exponentiated Weibull PDFs. It is shown, from the plots of the PDF and HRF of the BXW model, that this distribution is very flexible, accommodating a large number of shapes in the hazard function, such as “increasing”, “decreasing”, “upside down”, and “bathtub” (U-HRF). We derived explicit expressions for the ordinary and incomplete moments, quantile and generating functions, and moments of the residual life and reversed residual life model. We also obtained the PDFs of the order statistics and their moments. We discussed the estimation of the model parameters by maximum likelihood, along with numericak and graphical assessemnt via simulation studies. The model parameter was estimated by different methods of estimation, named the maximum likelihood method, the method of Cramer-Von-Mises estimation, the method of percentile estimation. And the method of L-moments. MCMC simulations and two applications were performed to compare the estimation methods. The proposed distribution was applied to two real data sets, which provides a better fit than several other competitive nested and non-nested models. We hope that the proposed model will attract wider application in areas such as engineering, survival, and lifetime data, meteorology, hydrology, economics, and others. Using the approach of the Bagdonavicius–Nikulin goodness-of-fit test for the right censored validation, we propose and apply a modified chi-square goodness-of-fit test for the BXW model. The modified goodness-of-fit statistic test is applied for a right censored real data set. Based on the censored maximum likelihood estimators on initial data, the modified goodness-of-fit test recovers the loss of information, while the grouped data follows the chi-square distribution. The elements of the modified criteria tests are derived. A real data application related to the laboratory investigation is for validation under the uncensored scheme.

## Figures and Tables

**Table 1:** Monte-Carlo simulation results: Average bias and MSE in parenthesis.

(θ,β)	θ	β
n = 100		
(0.4,2.5)	0.018 (0.018)	0.180 (0.946)
(3,0.2)	−0.114 (0.345)	0.008 (0.001)
(0.6,0.6)	0.002 (0.033)	0.053 (0.040)
(0.19,2.5)	0.038 (0.009)	−0.257 (0.449)
n = 200		
(0.4,2.5)	−0.004 (0.010)	0.180 (0.424)
(3,0.2)	−0.089 (0.172)	0.002 (5e^−4^)
(0.6,0.6)	−0.001 (0.019)	0.031 (0.018)
(0.19,2.5)	0.015 (0.004)	−0.206 (0.248)
n = 500		
(0.4,2.5)	0.002 (0.003)	0.036 (0.125)
(3,0.2)	−0.026 (0.068)	−0.002 (3e^−4^)
(0.6,0.6)	0.001 (0.007)	0.008 (0.005)
(0.19,2.5)	0.006 (0.002)	−0.164 (0.141)

**Table 2:** Average values (AVs) and the corresponding MSEs for n = 20.

Parameters	MLE	CVM	PerEs	L-Moment
Θ = 2	2.123510	2.088200	2.043230	2.09791
	(0.08387)	(0.32986)	(0.27218)	(0.35417)
Β = 0.5	0.51657	0.51770	0.50701	0.517620
	(0.00877)	(0.06045)	(0.01224)	(0.01580)
Θ = 0.6	0.64940	0.63391	0.66041	0.620200
	(0.02499)	(0.03179)	(0.05275)	(0.06030)
Β = 0.4	0.413460	0.41341	0.432070	0.402780
	(0.00592)	(0.00965)	(0.01652)	(0.01904)
Θ = 6	6.55660	6.251720	6.284390	6.695530
	(5.97827)	(2.75560)	(7.71943)	(23.64994)
Β = 0.1	0.103750	0.108850	0.095230	0.122000

**Table 3:** AVs and the corresponding MSEs for n = 50.

Parameters	MLE	CVM	PerEs	L-Moment
Θ = 2	2.04042	2.020530	1.9875600	2.028160
	(0.11254)	(0.11274)	(0.09788)	(0.13328)
Β = 0.5	0.50549	0.50440	0.49716	0.50573
	(0.00276)	(0.00471)	(0.00384)	(0.00685)
Θ = 0.6	0.61713	0.61232	0.62687	0.609540
	(0.00771)	(0.01104)	(0.01714)	(0.02187)
Β = 0.4	0.40422	0.40531	0.41483	0.40260
	(0.00216)	(0.00299)	(0.00533)	(0.00790)
Θ = 6	6.22870	6.14691	5.96776	6.212280
	(1.82638)	(1.04639)	(4.01295)	(8.14331)
Β = 0.1	0.10169	0.10262	0.09246	0.11227

**Table 4:** AVs and the corresponding MSEs for n = 150.

Parameters	MLE	CVM	PerEs	L-Moment
Θ = 2	2.00870	2.01118	1.99028	2.01254
	(0.03709)	(0.03613)	(0.03488)	(0.03911)
Β = 0.5	0.50107	0.50228	0.49783	0.50274
	(0.00095)	(0.00149)	(0.00124)	(0.00198)
Θ = 0.6	0.60668	0.60501	0.60609	0.60683
	(0.00262)	(0.00312)	(0.00607)	(0.00641)
Β = 0.4	0.40272	0.40228	0.40332	0.40313
	(0.00072)	(0.00086)	(0.00179)	(0.00236)
Θ = 6	6.08709	6.00740	5.75105	6.08802
	(0.49247)	(0.28755)	(2.26499)	(2.31531)
Β = 0.1	0.10068	0.10020	0.09448	0.10431

**Table 5:** AVs and the corresponding MSEs for n = 300.

Parameters	MLE	CVM	PerEs	L-Moment
Θ = 2	2.01328	2.01143	1.99373	2.00475
	(0.01739)	(0.01740)	(0.01622)	(0.01930)
Β = 0.5	0.50197	0.50233	0.49867	0.50085
	(0.00045)	(0.00071)	(0.00056)	(0.00101)
Θ = 0.6	0.60395	0.60365	0.60186	0.60209
	(0.00134)	(0.00164)	(0.00271)	(0.00311)
Β = 0.4	0.40181	0.40173	0.40109	0.40074
	(0.00036)	(0.00046)	(0.00077)	(0.00115)
θ = 6	6.02037	6.02330	5.56693	6.05655
	(0.25345)	(0.15614)	(1.65906)	(0.94734)
β = 0.1	0.10015	0.10042	0.09423	0.101650

**Table 6:** Parameter estimates and standard deviation in parenthesis for the first dataset.

Model	Estimates	Log-Likelihood
**BXW** (θ, β)	**40.768 (7.32) 0.095 (10 × e^−3^)**	**73.565**
Weibull (α, β)	1.227 (0.160) 4.557 (0.666)	74.788
Gamma (α, λ)	1.487 (0.184) 0.350 (0.051)	74.459
GE (α, λ)	1.560 (0.280) 0.309 (0.045)	74.396
EG (λ, p)	0.234 (0.042) 0.010 (0.280)	75.802
EP (λ, β)	0.011 (0.622) 0.235 (0.042)	75.795
CEG (λ, θ)	0.297 (0.047) 0.618 (0.190)	75.454

**Table 7:** Parameter estimates and standard deviation in parenthesis for second dataset.

Model	Estimates	Log-Likelihood
**BXW** (θ, β)	**14.347 (2.46) 0.104 (90.01)**	**55.049**
Weibull (α, β)	1.010 (0.125) 1.887 (0.320)	55.449
Gamma (α, λ)	1.062 (0.139) 0.565 (0.094)	55.413
GE (α, λ)	1.076 (0.184) 0.558 (0.092)	55.401
EG (λ, p)	0.481 (0.086) 0.177 (0.242)	55.395
EP (λ, β)	0.427 (0.596) 0.476 (0.085)	55.392
CEG (λ, θ)	0.532 (0.091) 0.999 (0.289)	55.453

**Table 8:** Formal goodness of fit statistics for the first dataset.

Model	Goodness of Fit Criteria					
	AIC	BIC	HQIC	CAIC	$W^{*}$	$A^{*}$
**BXW**	**151.131**	**153.999**	**152.066**	**151.559**	**0.061**	**0.395**
Weibull	153.577	156.445	154.512	154.006	0.118	0.713
Gamma	152.918	155.786	153.853	153.347	0.122	0.713
GE	152.793	155.661	153.728	153.222	0.120	0.705
EG	155.604	158.472	156.539	156.032	0.095	0.751
EP	155.590	158.458	156.525	156.019	0.095	0.749

**Table 9:** Formal goodness of fit statistics for the second dataset.

Model	Goodness of Fit Criteria					
	AIC	BIC	HQIC	CAIC	$W^{*}$	$A^{*}$
**BXW**	**114.098**	**117.151**	**115.139**	**114.485**	**0.032**	**0.227**
Weibull	114.899	117.952	115.940	115.286	0.043	0.282
Gamma	114.826	117.879	115.867	115.213	0.050	0.312
GE	114.803	117.856	115.844	115.190	0.052	0.317
EG	114.791	117.844	115.832	115.178	0.032	0.240
EP	114.785	117.837	115.826	115.172	0.032	0.239

**Table 10:** The values of estimators. $W^{*}$ and $A^{*}$ for all methods for the first data.

Method	θ	β	$W^{*}$	$A^{*}$
ML	40.768	0.095	0.05782	0.37572
CVM	35.997	0.087	0.05909	0.38163
**PerEs**	**55.730**	**0.101**	**0.05551**	**0.36612**
L-moment	42.097	0.098	0.05747	0.37407

**Table 11:** The values of estimators. $W^{*}$ and $A^{*}$ for all methods for the second data.

Method	θ	β	$W^{*}$	$A^{*}$
ML	14.347	0.105	0.02847	0.20972
CVM	17.784	0.097	0.02876	0.21271
PerEs	16.690	0.106	0.02910	0.21539
**L-moment**	**14.672**	**0.109**	**0.02846**	**0.20934**

**Table 12:** Biases and MSEs.

N = 10,000	n₁ = 20	n₂ = 50	n₃ = 150	n₄ = 300
θ = 1.5	1.4838 (0.0076)	1.4884 (0.0062)	1.4922 (0.0045)	1.4983 (0.0023)
β = 0.7	0.7192 (0.0089)	0.7137 (0.0077)	0.7096 (0.0057)	0.7043 (0.0034)
θ = 0.8	0.8213 (0.0082)	0.8126 (0.0058)	0.8084 (0.0032)	0.8012 (0.0016)
β = 0.5	0.4828 (0.0076)	0.4877 (0.0052)	0.4912 (0.0037)	0.4996 (0.0018)
θ = 3	2.9696 (0.0094)	2.9776 (0.0066)	2.9894 (0.0042)	2.9982 (0.0027)
β = 0.4	0.4331 (0.0068)	0.4284 (0.0044)	0.4167 (0.0029)	0.4024 (0.0013)

**Table 13:** Simulated levels of significance for the $Y_{n}^{2}\left(\phi \right)$ test for the BXW model against their theoretical values (ε = 0.01, 0.05, 0.10).

N = 10,000	n = 20	n = 50	n = 150	n = 300
ε = 1%	0.0055	0.0064	0.0085	0.0094
ε = 5%	0.0443	0.0452	0.0468	0.0486
ε = 10%	0.0931	0.0943	0.0959	0.0974

**Table 14:** Values of ${\hat{a}}_{j},e_{j},U_{j},{\hat{C}}_{1j},\mathrm{and}\;{\hat{C}}_{2j}$.

${\hat{a}}_{j}$	189.6	214.9	237.7	304
$U_{J}$	4	5	6	4
${\hat{C}}_{1j}$	0.9463	1.2416	0.8863	0.7648
${\hat{C}}_{2j}$	1.1346	0.9946	1.2476	0.9263
$e_{j}$	0.4859	0.4859	0.4859	0.4859
